# Heterogeneous liver uptake of Tc-99m-GSA as quantified through SPECT/CT helps to evaluate the degree of liver fibrosis: A retrospective observational study

**DOI:** 10.1097/MD.0000000000011765

**Published:** 2018-08-03

**Authors:** Kohei Kotani, Joji Kawabe, Shigeaki Higashiyama, Atsushi Yoshida, Etsushi Kawamura, Akihiro Tamori, Susumu Shiomi, Norifumi Kawada

**Affiliations:** a Department of Hepatology, Graduate School of Medicine; b Department of Nuclear Medicine, Graduate School of Medicine, Osaka City University; c Department of Gastroenterology and Hepatology, Osaka City Juso Hospital; d Department of Gastroenterology and Hepatology, Izumiotsu Municipal Hospital, Osaka, Japan.

**Keywords:** chronic hepatobiliary disease, liver fibrosis, scintigraphy, SPECT/CT, Tc-99m-GSA

## Abstract

Tc-99m-galactosyl human serum albumin (GSA) scintigraphy is used to assess the hepatic functional reserve, and allows for visual assessment of the residual hepatocyte distribution on single-photon emission computed tomography/computed tomography (SPECT/CT) images. The association between heterogeneous liver uptake of Tc-99m-GSA and liver fibrosis remains to be studied in detail. We analyzed this association.

Fifty-one patients with chronic hepatobiliary disease undergoing a Tc-99m-GSA scintigraphy were included in this study. The receptor (LHL15) and blood clearance (HH15) indexes (the uptake ratios of the liver and heart) were obtained from dynamic planar images. The liver uptake count maximum-to-mean ratio (LUC Max/Mean) was calculated from single-photon emission computed tomography/computed tomography (SPECT/CT) images as an indicator of the Tc-99m-GSA liver uptake heterogeneity. We assessed the relationship between these quantified values and liver fibrosis.

There were 30 Child-Pugh classification grade A patients, 16 grade B patients, and 5 grade C patients. Among the 30 patients whose liver histopathology was evaluable, those with advanced liver fibrosis (F2-4) had a lower LHL15 than those with mild liver fibrosis (F0-1) (median, 0.90 vs. 0.92, *P* = .04), and a higher LUC Max/Mean (median, 1.80 vs. 1.70, *P* = .02). The multivariate analysis identified platelets (*P* = .04) and the LUC Max/Mean (*P* = .04) as contributing factors of advanced liver fibrosis.

These findings suggest that Tc-99m-GSA SPECT/CT can be used not only to assess the hepatic functional reserve, but also to evaluate a degree of liver fibrosis.

## 1 Introduction

Objective assessment of the hepatic functional reserve of patients with chronic liver disease can be difficult. In daily clinical practice, the scores and grades of the Child-Pugh classification^[1]^ and the model for end-stage liver disease (MELD)^[2]^ are commonly used. Although these values can be easily calculated from blood test data, they are affected by various factors, including malnutrition, medication history, and biliary stenosis. Therefore, it is preferable to use a method for assessment of the hepatic functional reserve that is less likely to be affected by factors external to the liver.

In mammals, asialoglycoprotein receptors are expressed on the surface of normal hepatocytes.^[3,4]^ The number of receptors is known to be reduced by various liver disorders, and is reflected by the number of residual hepatocytes.^[5–7]^ As galactosyl human serum albumin (GSA) specifically binds to the asialoglycoprotein receptors, Tc-99m-GSA scintigraphy reflects the residual hepatocyte function and is less likely to be affected by extra-hepatic factors. Previous reports have demonstrated the usefulness of Tc-99m-GSA scintigraphy for assessment of the hepatic functional reserve of patients with chronic liver disease. However, these reports focused on the use of indicators obtained from planar images, such as the hepatic receptor index (LHL15) and the blood clearance index (HH15).^[8–11]^

Single-photon emission computed tomography/computed tomography (SPECT/CT) is an imaging modality that combines a gamma camera with CT, allowing the patient to be imaged in the same position on a single bed. This enables the construction of images without anatomical misalignment by combining the functional images obtained from the SPECT with the morphological images obtained from the CT. Furthermore, CT images are used for attenuation correction of the SPECT images, thereby increasing the latter's quality. Therefore, SPECT/CT allows for a more accurate quantitative assessment than SPECT alone or the fusion images of SPECT with different phase CT images.^[12–14]^ As the combined use of SPECT and CT in Tc-99m-GSA scintigraphy enables a regional assessment of the hepatocyte function and radioisotope (RI) volumetry, it has been applied to the assessment of various hepatic diseases.^[15–20]^

Although liver biopsy is the criterion standard for the diagnosis of liver fibrosis complicating chronic liver disease, it is an invasive procedure with the risk of serious complications such as massive bleeding, bile peritonitis, or visceral perforation. Recently, the usefulness of various biomarkers and diagnostic imaging modaities for the noninvasive diagnosis of this type of liver fibrosis has been reported.^[21–27]^ An association between functional indices such as the LHL15 or the HH15 obtained from Tc-99m-GSA scintigraphy planar images and the staging of liver damage has been reported^[8,28]^; however, no previous report has described the association between liver heterogeneity of Tc-99m-GSA accumulation in SPECT/CT images and liver fibrosis.

Therefore, this study aimed to investigate whether heterogeneous liver uptake of Tc-99m-GSA as quantified through SPECT/CT can contribute to the assessment of the hepatic functional reserve and the prediction of liver fibrosis in patients with chronic hepatobiliary disease.

## 2 Methods

### 2.1 Patients

Fifty-one patients with chronic hepatobiliary disease who had undergone a Tc-99m-GSA scintigraphy combined with a SPECT/CT between April 2010 and May 2016 were included in this study. Patients with neoplastic disease in both hepatic lobes or with post percutaneous transhepatic portal embolization were excluded. The patients’ characteristics are presented in Table 1. The study population comprised 28 men and 23 women, with a median age of 66 years. The hepatic diseases included the following: primary liver carcinoma (13 patients), metastatic liver carcinoma (12 patients), diffuse liver disease (12 patients), and others—including cholangiocarcinoma, other malignant neoplastic non-hepatic diseases, and cholangitis—(14 patients). Twenty-seven patients (53%) had underlying liver diseases, including chronic hepatitis C in 12 patients, alcoholic liver disease (ALD), or non-alcoholic fatty liver disease (NAFLD) in 9 patients, and other diseases, such as autoimmune hepatitis or chronic hepatitis B, in 6 patients. Thirty patients (59%) received a pathological diagnosis. Based on the meta-analysis of histological data in viral hepatitis (METAVIR) scores for the assessment of liver fibrosis (F0, no fibrosis; F1, portal fibrosis without septa; F2, portal fibrosis and few septa; F3, numerous septa without cirrhosis; and F4, cirrhosis),^[29,30]^ 20 patients had no or mild liver fibrosis (F0-1), whereas 10 had advanced liver fibrosis (F2-4). Before performing Tc-99m-GSA scintigraphy, the purpose of this diagnostic tool was explained to each patient and oral informed consent was obtained. This study complied with the ethical guidelines of the 1964 Declaration of Helsinki (2008 revision) and was conducted with the approval of the Institutional Review Board in Osaka City University Hospital (approval number 3461). Because this study was conducted as a retrospective observational design, opt-out methodology was applied. We provided patients an opportunity for opting out of the study on our institution's homepage.

**Table 1:**
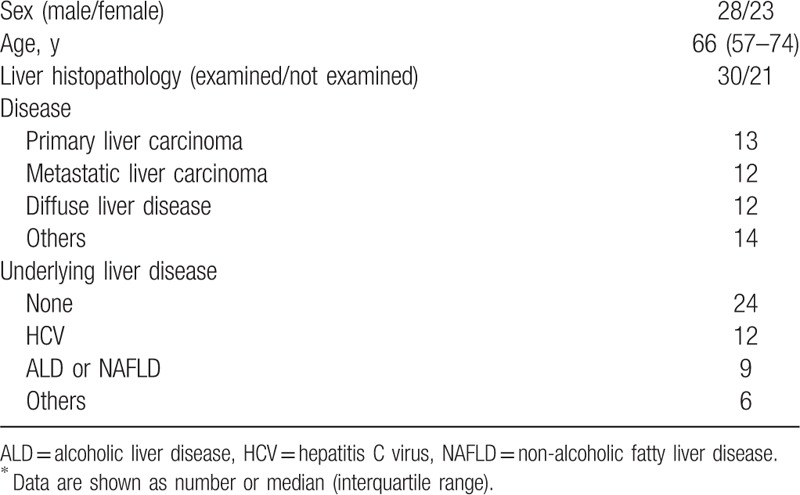
Patients’ clinical characteristics (n = 51)^∗^.

### 2.2 Conditions of data collection and Tc-99m-GSA scintigraphy image construction

This study used the Brightview X system (Philips Medical Systems Inc., Cleveland, OH) imaging device. The latter is equipped with a dual detector gamma camera incorporating a flat-panel cone-beam CT system that allows for SPECT and CT to be performed without changing the patient's position, and for the accurate attenuation correction of SPECT images with CT.^[31]^ A 185-MBq dose of Tc-99m-GSA (Nihon Medi-Physics Co. Ltd., Tokyo, Japan) was injected through the patients’ cubital vein, and planar images were dynamically acquired immediately after the injection in frames of 60 seconds for 20 minutes. For the planar images, the data were collected in a 256 × 256 pixel matrix size with a low-energy, high-resolution (LEHR) collimator. The SPECT was initiated 20 minutes after the venous injection. The SPECT data were collected in a 128 × 128 pixel matrix size using the LEHR collimator. They were acquired for a total of 32 minutes (1 step, 30 seconds; step angle, 5.63°; 64 steps, 360°), and were reconstructed using the Astonish method (2 iterations, 16 subsets). The attenuation and scatter corrections of the SPECT images were performed with cone-beam CT images. Hanning filtering (cut-off: 1.5 cycles per pixel) was used in the filtering process. The reconstructed SPECT images and CT images were converted to the digital imaging and communications in medicine (DICOM) format, and were transferred to an extended brilliance workspace-nuclear medicine (EBW-NM) workstation (Philips Medical Systems Inc., Cleveland, OH). The images were constructed and analyzed using the asialo analysis of the interface definition language (IDL) sample application software version 2.0.1 supplied by the Hitachi Medical Corporation (Tokyo, Japan), whereas the SPECT/CT data were analyzed using the volume analysis of the software.

### 2.3 Analysis of planar images

Dynamic acquisition was performed for 20 minutes after the venous RI injection. Regions of interest (ROIs) were defined in the liver and heart on the 20-minute integrated images, and time activity curves were generated for each ROI. According to the previously reported method, the LHL15 and HH15 were determined as follows: LHL15, liver RI count at 15 minutes/(liver RI count + heart RI count at 15 minutes); HH15, heart RI count at 15 min/heart RI count at 3 minutes.^[8,9]^

### 2.4 Analysis of SPECT/CT images

**Figure 1:**
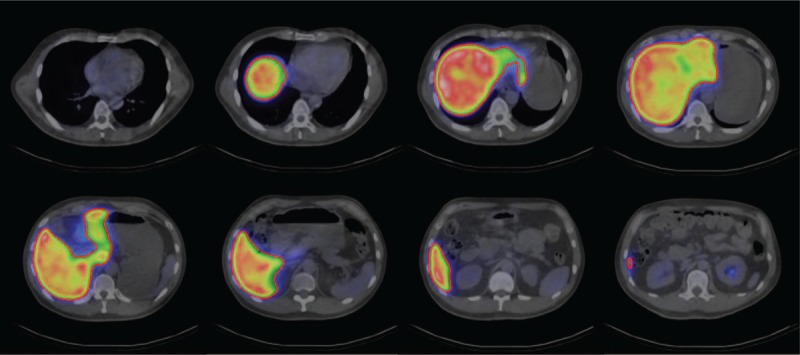
Quantitative evaluation of single-photon emission computed tomography/computed tomography (SPECT/CT) images. A region of interest (ROI) was defined in the liver for each cross-sectional SPECT/CT image of the Tc-99m-GSA scintigraphy. The three-dimensional volume of interest (VOI) was determined by adding up the ROI data obtained from all cross-sectional images. The maximum value and mean value per voxel of the liver VOI were calculated. The liver uptake count maximum-to-mean ratio (LUC Max/Mean) was then calculated and used as an indicator of the liver uptake heterogeneity.

An ROI was defined in the liver on each cross-sectional SPECT/CT image. The three-dimensional volume of interest (VOI) was determined by combining all cross-sectional images (Fig. 1). The maximum value and mean value per voxel of the liver VOI were calculated, followed by the liver uptake count maximum-to-mean ratio (LUC Max/Mean). We newly defined this LUC Max/Mean value as an indicator of liver uptake heterogeneity of Tc-99m-GSA. It means that liver uptake of Tc-99m-GSA is homogeneous if LUC Max/Mean value is close to 1; on the other hand, liver uptake of Tc-99m-GSA is heterogeneous when LUC Max/Mean value is away from 1. For patients with diffuse liver disease, images of the whole liver were analyzed, whereas the analysis was limited to images of the lobe without any tumor in patients with liver tumors. The right and left lobes were divided by Cantlie line. Furthermore, the CT images were used to determine the presence or absence of ascites and the maximum spleen diameter.

### 2.5 Statistical analysis

The JMP 10.0.0 statistical software (SAS Institute Inc., Cary, NC) was used for the statistical analysis. The continuous variables were expressed as medians (interquartile range). The correlation between the Child-Pugh scores and the scintigraphic data were analyzed with the Spearman rank correlation coefficient (ρ). To assess the differences in scintigraphic data according to the etiology of the liver disorders, a Kruskal-Wallis rank sum test was performed by dividing the patients with underlying liver diseases into three groups: a group for patients with hepatitis C virus (HCV) infections, 1 for patients with ALD or NAFLD, and 1 for patients with other liver diseases. Scintigraphic data were compared between patients with no or mild liver fibrosis (F0-1) and patients with advanced liver fibrosis (F2-4) by the Mann-Whitney-Wilcoxon test. To assess the diagnostic value for liver fibrosis, univariate and multivariate logistic regression analyses were used to identify the factors that could contribute to the diagnosis of advanced liver fibrosis. Furthermore, a receiver-operating characteristic (ROC) analysis was performed to determine the thresholds at which the contributing factors provided the best diagnostic value. A *P* value <.05 was considered to denote statistical significance.

## 3 Results

### 3.1 Patients’ laboratory and scintigraphic data

**Figure 2:**
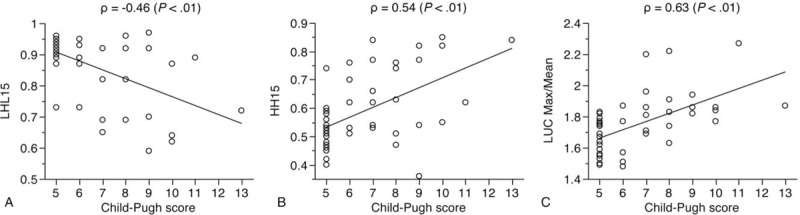
Correlation between scintigraphic data and Child-Pugh scores. The Child-Pugh score was negatively correlated with the LHL15 (A: ρ = −0.46, *P* < .01) and positively correlated with the HH15 (B: ρ = 0.54, *P* < .01) and the LUC Max/Mean (C: ρ = 0.63, *P* < .01).

The laboratory and scintigraphic data for all patients are shown in Table 2. The median Child-Pugh score was 6, including 30 grade A patients, 16 grade B patients, and 5 grade C patients. The median LHL15 was 0.91, HH15 was 0.55, and LUC Max/Mean was 1.75. The correlations between the Child-Pugh scores and the scintigraphic data are presented in Figure 2. The LHL15 showed a significantly negative correlation with the Child-Pugh scores (ρ = −0.46, *P* < .01), whereas the HH15 (ρ = 0.54, *P* < .01) and the LUC Max/Mean (ρ = 0.63, *P* < .01) showed a significant positive correlation with these scores.

**Table 2:**
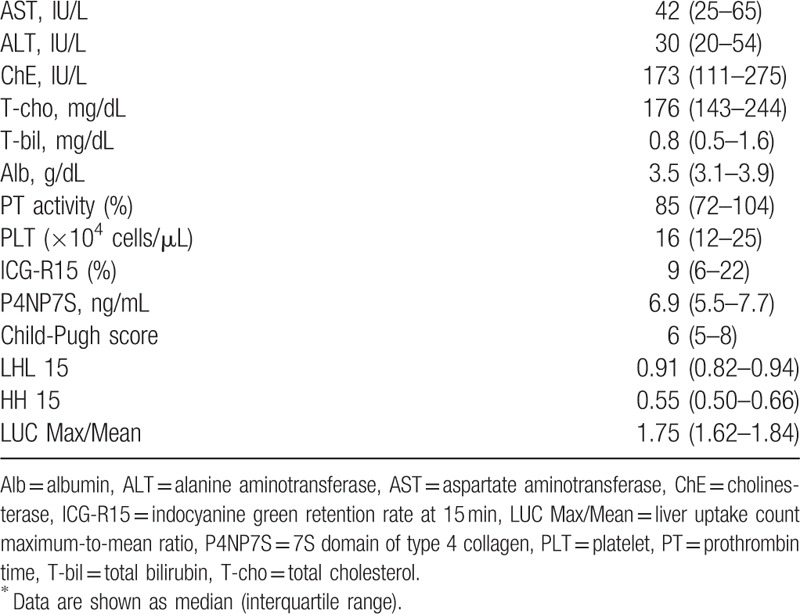
Laboratory tests and scintigraphic data of all patients (n = 51)^∗^.

### 3.2 Scintigraphic data comparison according to etiology of underlying liver diseases

The Tc-99m-GSA scintigraphic data of the 27 patients with underlying liver diseases were compared by etiology (Table 3). No significant differences were observed in the distributions of the LHL15, the HH15, and the LUC Max/Mean (*P* = .91, .96 and .36, respectively).

**Table 3:**
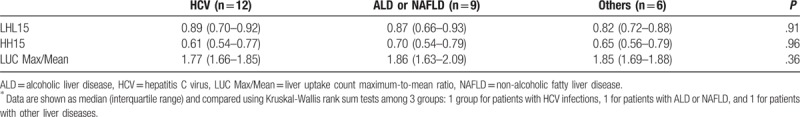
Scintigraphic data of patients with underlying liver disease (n = 27)^∗^.

### 3.3 Scintigraphic data comparison between patients with no or mild liver fibrosis and patients with advanced liver fibrosis

**Figure 3:**
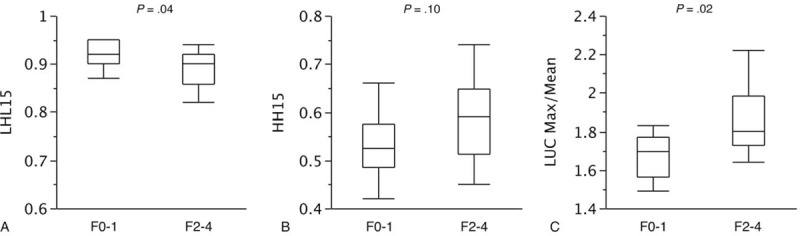
Scintigraphic data of patients with no or mild liver fibrosis (F0-1) and of patients with advanced liver fibrosis (F2-4). The F2-4 group showed a lower LHL15 than the F0-1 group (A: median, 0.90 [interquartile range, IQR, 0.86–0.92] vs. 0.92 [IQR, 0.90–0.95], *P* = .04) and a higher LUC Max/Mean (B: median, 1.80 [IQR, 1.73–1.98] vs. 1.70 [IQR, 1.56–1.77], *P* = .02). No significant difference in HH15 was observed between the 2 groups (C: median, 1.53 [IQR, 0.49–0.58] vs. 0.59 [IQR, 0.51–0.65], *P* = .10).

The scintigraphic data of the F0-1 (no or mild liver fibrosis) and F2-4 (advanced liver fibrosis) groups are provided in Figure 3. The LHL15 was lower in the F2-4 group than in the F0-1 group (median, 0.90 [interquartile range, IQR, 0.86−0.92] vs. 0.92 [IQR, 0.90−0.95], *P* = .04), whereas the LUC Max/Mean was higher in the F2-4 group than in the F0-1 group (median, 1.80 [IQR, 1.73−1.98] vs. 1.70 [IQR, 1.56−1.77], *P* = .02).

### 3.4 Factors contributing to the prediction of advanced liver fibrosis

The results of the univariate and multivariate logistic regression analyses for identification of the factors contributing to the prediction of advanced liver fibrosis are shown in Table 4. The univariate analysis identified the spleen size, T-cho level, PT, PLT count, LHL15, and LUC Max/Mean as significant factors. In a multivariate analysis including these factors, the PLT count (odds ratio [OR], 0.78, 95% confidence interval [CI], 0.36−0.99, *P* = .04) and the LUC Max/Mean (OR, 1.22, 95% CI, 1.01−2.31, *P* = .04) were identified as independent contributing factors to the prediction of advanced liver fibrosis. The optimal cutoff values for the prediction of advanced liver fibrosis were 15 × 10^4^ cells/μL for the PLT count (sensitivity 80%, specificity 85%), and 1.83 for the LUC Max/Mean (sensitivity 50%, specificity 95%) in ROC analyses.

**Table 4:**
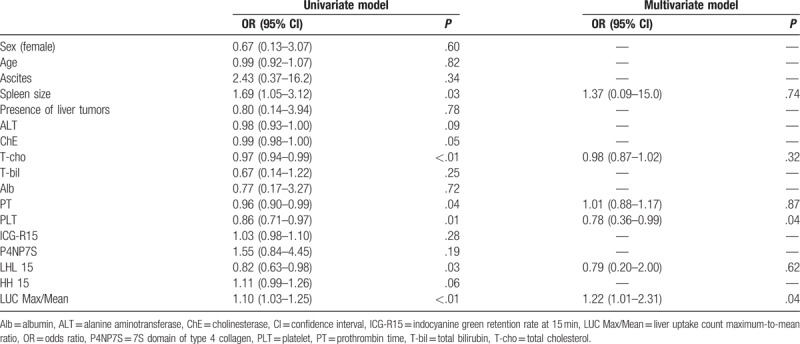
Results of univariate and multivariate logistic regression analysis for advanced liver fibrosis.

## 4 Discussion

The hepatic functional reserve is commonly assessed with blood test variables that reflect the liver synthetic capacity (such as the serum albumin and prothrombin time), Child-Pugh scores based on a range of variables (including the above), and the MELD score in the presence of end-stage cirrhosis. However, these indicators are often unreliable, as they are affected by various factors (e.g., undernutrition, the administration of blood preparations and other drugs, and biliary stenosis). The indocyanine green (ICG) test is a load test for the assessment of the hepatic functional reserve that measures the percentage of the residual ICG in the blood 15 minutes after venous injection (R15), as the ICG is taken up by the hepatocytes and excreted in the bile without conjugation. However, this test is unreliable for patients with biliary stenosis or portosystemic shunts.^[32]^ As the GSA binds to the asialoglycoprotein receptors, which are specifically expressed on the hepatocytes, Tc-99m-GSA scanning is a form of receptor scintigraphy that can be regarded as a method for assessment of the hepatic functional reserve less likely to be affected by extrahepatic factors.

Echoing previous findings,^[8]^ this study revealed that the LHL15 and HH15 were correlated with the Child-Pugh scores, thereby suggesting that Tc-99m-GSA scintigraphy could contribute to the assessment of the hepatic functional reserve. Moreover, the LUC Max/Mean was also correlated with the Child-Pugh scores, suggesting that the hepatocytes became heterogeneously damaged as the hepatic functional reserve decreased. Patients with advanced liver fibrosis often have a poor hepatic functional reserve; the risk of carcinogenesis of hepatocellular carcinoma increases in these patients. This study revealed that patients with advanced liver fibrosis had a lower LHL15 and a higher HH15 than those with no or mild liver fibrosis. Although LHL15 and HH15 are suitable for assessment of the hepatic functional reserve, they only reflect the rate of RI binding to the receptors and the blood clearance rate. These indices serve as indirect indicators of the liver fibrosis severity. Therefore, a more direct assessment based on SPECT images is desirable. The distribution of the liver RI uptake can be visually assessed from Tc-99m-GSA SPECT images. However, it has been suggested that SPECT data are not very reliable, and SPECT presents the disadvantage of making quantitative assessment difficult, as the gamma rays are absorbed and scattered in the patient's body. Through the attenuation and scatter correction of SPECT images with CT images, the SPECT/CT system can produce high-quality SPECT images that are visually superior in terms of space resolution. In addition, the system is also more reliable for quantitative assessment.^[12,13]^

Although there have been sporadic reports of the association between Tc-99m-GSA scintigraphic images and liver fibrosis,^[8,28]^ few have discussed the association between SPECT/CT images and liver fibrosis.^[18]^ Yoshida et al compared the functional liver index (FLI) as an indicator of the radioactivity for hepatocytes per liver volume on SPECT/CT images with other liver fibrosis markers, and reported that the FLI was most useful for the diagnosis of severe (F3 or higher) liver fibrosis.^[18]^ The texture of the liver parenchyma is known to become coarser as the liver fibrosis progresses.^[33–37]^ To the best of our knowledge, this was the first study to use SPECT/CT to determine the association between the heterogeneous uptake of Tc-99m-GSA and liver fibrosis. In the present study, we found that SPECT/CT could be used to diagnose advanced liver fibrosis (F2-4) associated with a high LUC Max/Mean, that is, a highly heterogeneous uptake of Tc-99m-GSA. The portal area of patients with chronic liver disease becomes more fibrotic and extended than that of normal individuals. Although the Tc-99m-GSA uptake level is maintained in areas with a relatively large number of hepatocytes in the lobules, it decreases in close proximity to the extended portal area. Therefore, the overall mean intensity of the Tc-99m-GSA uptake in the liver is likely to decrease as a result. For these reasons, the LUC Max/Mean seemed to be higher in patients with advancing liver fibrosis, although this opinion is a theory and requires further validation. In this study, the cutoff PLT count for the diagnosis of advanced liver fibrosis was identified as 15 × 10^4^ cells/μL by an ROC analysis. It is well-known that many chronic hepatitis C patients with a PLT count of 15 × 10^4^ cells/μL or less have F2 or greater liver fibrosis.^[38,39]^ Our results seemed appropriate in comparison with previously reported values.

Besides Tc-99m-GSA SPECT/CT method, ultrasonographic shear-wave elastography (SWE) such as Fibroscan and magnetic resonance elastography (MRE) are known as the examinations for noninvasively predicting liver fibrosis. SWE can be performed most conveniently among these modalities, whereas the skill of the operator is necessary; especially in obese patients, the success rate is often low. MRE can be reliably performed even in obese patients and those with ascites on the liver surface, but measured value is affected by inflammation and congestion as with SWE. Tc-99m-GSA SPECT/CT method we examined in this study evaluates the heterogeneity of hepatocyte distribution. Although it differs from these elastography methods that use the propagation velocity of mechanical wave in the body, it can be performed in obese patients and those with ascites, and measured value is not affected by inflammation and congestion. Furthermore, this method has an advantage that it can evaluate not only fibrosis of the whole liver but also the hepatic functional reserve.

This study had some limitations. First, as this study was conducted as a retrospective observational design, it was difficult to compare SPECT/CT data with other methods for liver fibrosis prediction such as ultrasonography or magnetic resonance imaging. Second, the statistical power may have been low because of the small sample size. Third, there may have been a population bias as the study was conducted as a single-center study. Inflammation has been reported to occur heterogeneously in the liver in some cases of autoimmune hepatitis.^[40,41]^ This study found no differences according to the etiology of the liver disease. This result may be explained by the relatively large number of patients with HCV infection as compared with the very small of number of those with autoimmune hepatitis. Further validation studies with larger patient cohorts should be conducted to compare the scintigraphic data according to etiology of liver disease.

## 5 Conclusion

We demonstrated that the LUC Max/Mean obtained from SPECT/CT images could serve as a noninvasive and quantitative index of the Tc-99m-GSA liver uptake heterogeneity. Similarly to the LHL15 and HH15, the LUC Max/Mean was significantly correlated with the Child-Pugh score. A multivariate logistic regression analysis identified the platelet count and the LUC Max/Mean as contributing factors to the prediction of advanced liver fibrosis. Therefore, Tc-99m-GSA scintigraphy combined with SPECT/CT is a useful modality for the assessment of the hepatic functional reserve and the degree of liver fibrosis in patients with chronic hepatobiliary disease.

## Acknowledgments

The authors sincerely thank Mr. Yutaka Katayama, and Mr. Takashi Yamanaga for providing technical cooperation.

## Author contributions

**Conceptualization:** Kohei Kotani, Etsushi Kawamura, Susumu Shiomi.

**Data curation:** Kohei Kotani, Shigeaki Higashiyama, Atsushi Yoshida.

**Investigation:** Kohei Kotani, Shigeaki Higashiyama, Atsushi Yoshida.

**Methodology:** Joji Kawabe, Etsushi Kawamura, Susumu Shiomi.

**Supervision:** Joji Kawabe, Akihiro Tamori, Susumu Shiomi, Norifumi Kawada.

**Writing – original draft:** Kohei Kotani.

**Writing – review & editing:** Akihiro Tamori, Susumu Shiomi, Norifumi Kawada.
